# A pyridinium/anilinium [2]catenane that operates as an acid–base driven optical switch

**DOI:** 10.3762/bjoc.14.165

**Published:** 2018-07-25

**Authors:** Sarah J Vella, Stephen J Loeb

**Affiliations:** 1 Department of Chemistry and Biochemistry, University of Windsor, Windsor, Ontario N9B 3P4, Canada

**Keywords:** catenane, mechanically interlocked molecule, molecular switch

## Abstract

A two-station [2]catenane containing a large macrocycle with two different recognition sites, one bis(pyridinium)ethane and one benzylanilinium, as well as a smaller **DB24C8** ring was synthesized and characterized. ^1^H NMR spectroscopy showed that the **DB24C8** ring can shuttle between the two recognition sites depending on the protonation state of the larger macrocycle. When the aniline group is neutral, the **DB24C8** ring resides solely at the bis(pyridinium)ethane site, while addition of acid forms a charged benzylanilinium site. The **DB24C8** then shuttles between the two charged recognition sites with occupancy favoring the bis(pyridinium)ethane site by a ratio of 4:1. The unprotonated [2]catenane has a deep yellow/orange color when the **DB24C8** ring resides solely at the bis(pyridinium)ethane site and changes to colorless when the crown ether is shuttling (i.e., circumrotating) back and forth between the two recognition sites thus optically signalling the onset of the shuttling dynamics.

## Introduction

**Figure 1:**
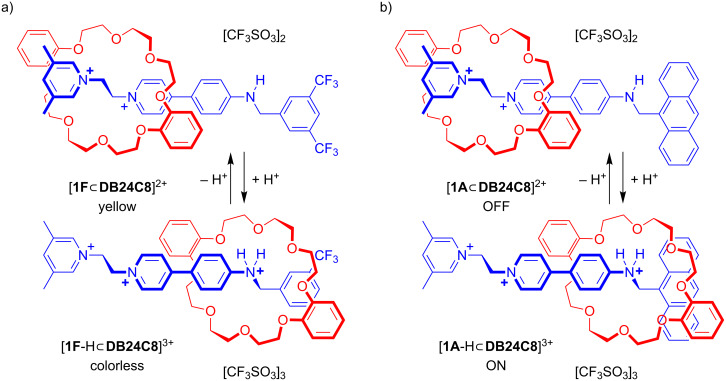
Two [2]rotaxane molecular shuttles with both bis(pyridinium)ethane and benzylanilinium recognition sites that can be switched by acid–base chemistry and optically sensed by a) a color change from colorless to yellow and b) a change in fluorescence from OFF to ON (CD_3_CN or CD_2_Cl_2_). Code: F to indicate CF_3_ groups; A to indicate anthracene group.

[2]Rotaxane molecular shuttles [1–5] are the dynamic building blocks of a wide variety of molecular switches [6–9] and a number of sophisticated molecular machines that operate away from equilibrium [10–15]. We have previously reported [2]rotaxane molecular switches containing a single dibenzo[24]crown ether **DB24C8** wheel and two different recognition sites; benzylanilinium and 1,2-bis(pyridinium)ethane [16]. These shuttles operate as bistable switches driven by acid/base chemistry and can be optically sensed by either a change in color (yellow/colorless) for [**1F**
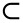
**DB24C8**]^2+^ or a fluorescence change (OFF/ON) for [**1A**
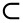
**DB24C8**]^2+^; see Figure 1.

**Figure 2:**
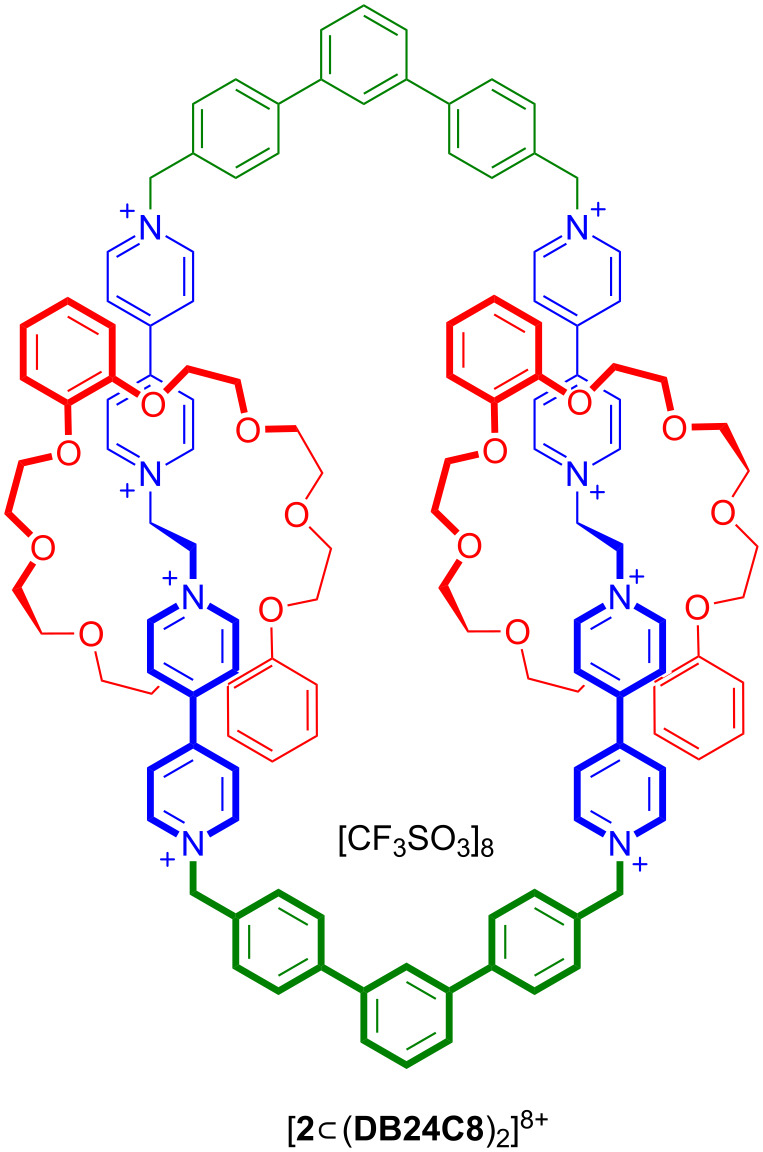
A [3]catenane containing two identical bis(pyridinium)ethane recognition sites on a large macrocycle and two smaller threaded **DB24C8** rings.

In addition, we have also previously prepared a [3]catenane containing two dibenzo[24]crown ether **DB24C8** rings interlocked onto a much larger macrocyclic ring containing two 1,2-bis(pyridinium)ethane recognition sites linked by terphenyl spacer groups [17] (Figure 2).

**Scheme 1:**
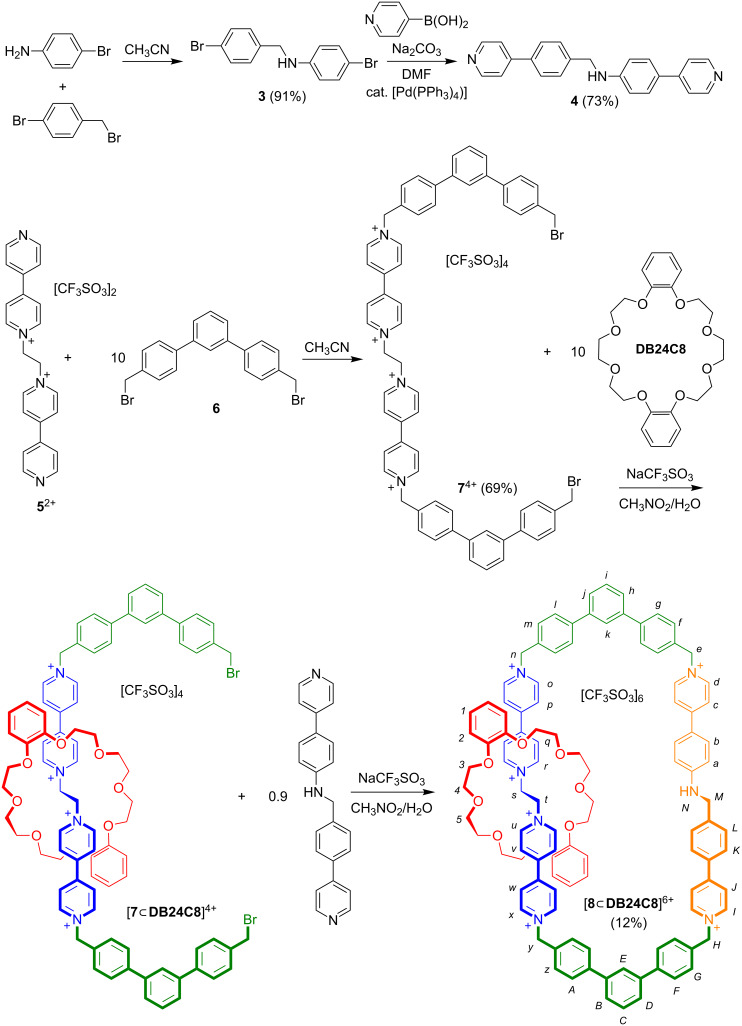
Step-wise synthesis of [2]catenane [**8**
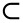
**DB24C8**]^6+^ containing benzylanilinium and bis(pyridinium)ethane recognition sites and terphenyl spacers.

It was thus of interest to design and build these two different recognition sites (benzylanilinium and bis(pyridinium)ethane) into an analogous circumrotational [2]catenane molecular switch to compare to the linear [2]rotaxane molecular shuttles outlined in Figure 1. This should be possible because of the structural similarities (size and shape) between the bis(pyridinium)ethane and benzylanilinium recognition sites. Each has a two-atom chain in a low energy, *anti*-conformation linking aromatic rings and the distance between the terminal nitrogen atoms are 18.11 and 18.09 Å (MM3) for the benzylaniline and bis(dipyridinium)ethane axles **4** and **5**^2+^, respectively; see Figure 2 and Scheme 1 compound [**8**
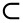
**DB24C8**]^6+^ for this comparison and concept.

## Results and Discussion

### Synthesis

Although the previously reported [3]catenane (Figure 2) was synthesized using a one-step, self-assembly procedure from two bis(pyridinium)ethane axles, two terphenyl spacers and two **DB24C8** crown ethers, a [2]catenane with different recognition sites requires a stepwise approach involving the incorporation of each recognition site independently. Overall, the synthesis of [2]catenane [**8**
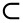
**DB24C8**]^6+^ required multiple steps and is outlined in Scheme 1. Two literature preparations were used to construct each of the known compounds, terphenyl linker **6** [18] and bis(pyridinium)ethane axle [**5**][OTf]_2_ [19–20], while the new benzylaniline axle **4** was prepared as shown from **3** [21]**.** Once the precursor components were synthesized, the [2]catenane was assembled in two steps. Firstly, [**5**][OTf]_2_ was reacted with ten equivalents of the bis(bromomethyl)terphenyl linker **6** in CH_3_CN to afford [**7**][OTf]_4_ in moderate yield. Secondly, the [2]pseudorotaxane [**7**
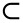
**DB24C8**]^4+^ was formed using [**7**][OTf]_4_ in the presence of **DB24C8** followed by ring closure using the benzylaniline axle **4** to yield [**8**
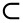
**DB24C8**][OTf]_6_. The reaction was performed under dilute conditions with 10 equivalents of crown ether to favor ring closure and kinetic trapping of the smaller ring.

To isolate the pure [2]catenane, the reaction solvent (CH_3_CN) was evaporated and the residue washed with toluene to remove excess crown ether. This was then followed by column chromatography on silica gel using a 5:3:2 mixture of CH_3_OH/2 M NH_4_Cl/CH_3_NO_2_ as the eluent. Fractions containing the product (*R*_f_ = 0.66) were combined and anion exchanged to the triflate salt to yield [2]catenane [**8**
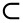
**DB24C8**][OTf]_6_.

### Characterization

**Figure 3:**
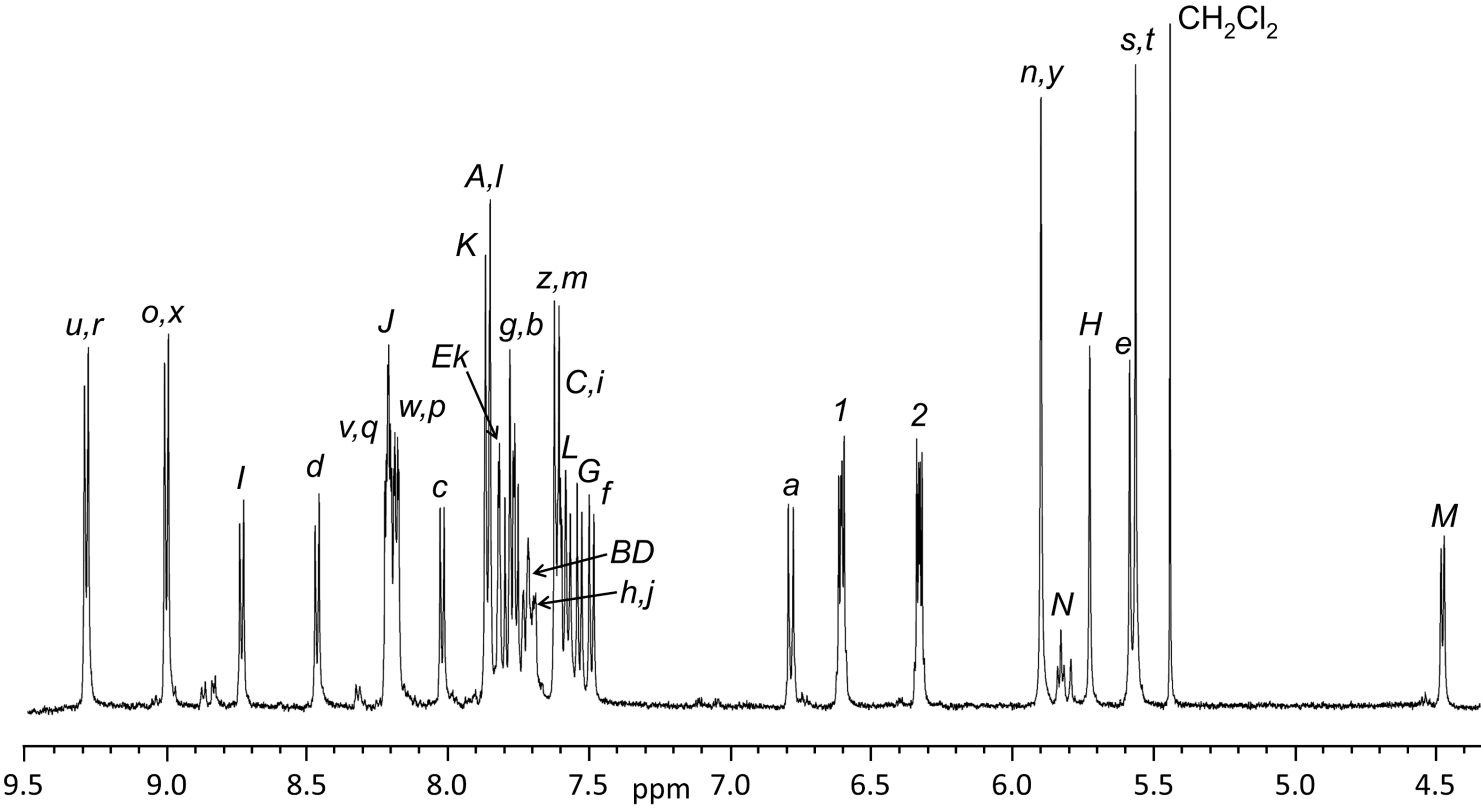
^1^H NMR spectrum of [2]catenane [**8**
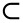
**DB24C8**]^6+^ (500 MHz, 298 K, CD_2_Cl_2_) showing the assigned proton chemical shifts; see Scheme 1 for labelling.

The ^1^H NMR spectrum of [2]catenane [**8**
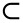
**DB24C8**]^6+^ (298 K, CD_2_Cl_2_) is shown in Figure 3 and the labelling scheme for the H-atoms is given in Scheme 1. All resonances were assigned based on 2D COSY NMR spectroscopy as well as comparison to ^1^H NMR and COSY spectra of individual components **6** and **7**^4+^. Comparing the proton chemicals shifts for H-atoms *n*–*y* of [**8**
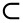
**DB24C8**]^6+^ with those of precursor **7**^4+^ shows changes in chemical shift typically associated with the close interaction of **DB24C8** with a bis(pyridinium)ethane recognition site [18]. In particular, the significant downfield shifts observed for ethylene protons *s* and *t* from 5.30 ppm in **7**^4+^ to 5.56 ppm for [**8**
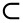
**DB24C8**]^6+^ as well as *u* and *r*, the *ortho* pyridinium protons, from 9.04 ppm in **7**^4+^ to 9.31 ppm for [**8**
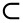
**DB24C8**]^6+^ are characteristic of hydrogen-bonding to the crown ether. In addition, π-stacking interactions induce upfield shifts for protons *p*, *q*, *v* and *w* from 8.48 ppm in **7**^4+^ to 8.24 ppm for [**8**
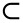
**DB24C8**]^6+^. Protons *o*, *x*, *n* and *y* do not shift appreciably because the crown ether does not extend far enough to interact with these protons. In contrast, the chemical shifts for protons *a*–*d* and *I*–*L* on the benzylaniline portion of the large ring of [**8**
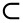
**DB24C8**]^6+^ do not shift significantly inferring that in the neutral aniline state the crown ether resides exclusively at the bis(pyridinium)ethane site of the [2]catenane. Table 1 summarizes the chemical shift differences between the [2]catenane [**8**
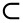
**DB24C8**]^6+^ and precursor **7**^4+^ which contains no crown ether.

**Table 1:** Summary of major chemical shift differences between precursor **7**^4+^ and catenane [**8**
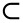
**DB24C8**]^6+^.

protons^a^	**7**^4+^	[**8** 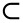 **DB24C8**]^6+^

*n, y*	5.89	5.90
*o, x*	9.05	9.03
*p, w*	8.47	8.19
*q, v*	8.50	8.22
*r, u*	9.04	9.31
*s, t*	5.30	5.56

^a^All chemical shift values given in ppm relative to TMS in CD_3_CN at 298 K.

A sample of [**8**
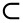
**DB24C8**]^6+^ (1:1 CH_3_OH/CH_3_CN) was analyzed by high-resolution electrospray mass spectrometry (HRESIMS). Sufficient resolution for each of the 2+, 3+, 4+ and 5+ molecular ions allowed for exact mass measurements (<5 ppm) confirming the catenated nature of the structure. Table 2 summarizes the observed values.

**Table 2:** Summary of major HRESIMS peaks for catenane [**8**
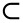
**DB24C8**]^6+^.

molecular ion	calculated *m*/z	experimental *m*/z	Δ (ppm)

{[**8** 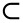 **DB24C8**][OTf]_4_}^2+^	1116.7969	1116.7972	0.3
{[**8** 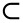 **DB24C8**][OTf]_3_}^3+^	694.8804	694.8835	4.5
{[**8** 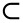 **DB24C8**][OTf]_2_}^4+^	483.9222	483.9246	5.0
{[**8** 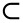 **DB24C8**][OTf]}^5+^	357.3472	357.3465	2.0

### Acid–base driven switching

The analysis of the ^1^H NMR spectrum (CD_3_CN, 298 K) of [**8**
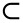
**DB24C8**]^6+^ indicates that the **DB24C8** ring resides exclusively at the bis(pyridinium)ethane recognition site. This is easily understood as the neutral benzylaniline site does not allow for appreciable non-covalent interactions and cannot compete for the **DB24C8** ring with the dicationic bis(pyridinium)ethane site. However, the addition of one equivalent of triflic acid (CF_3_SO_3_H) to a solution of [**8**
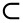
**DB24C8**]^6+^ results in protonation of the aniline nitrogen atom to give [8-**H**
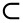
DB24C8]**^7^**^+^ and a second viable recognition site for the crown ether.

**Figure 4:**
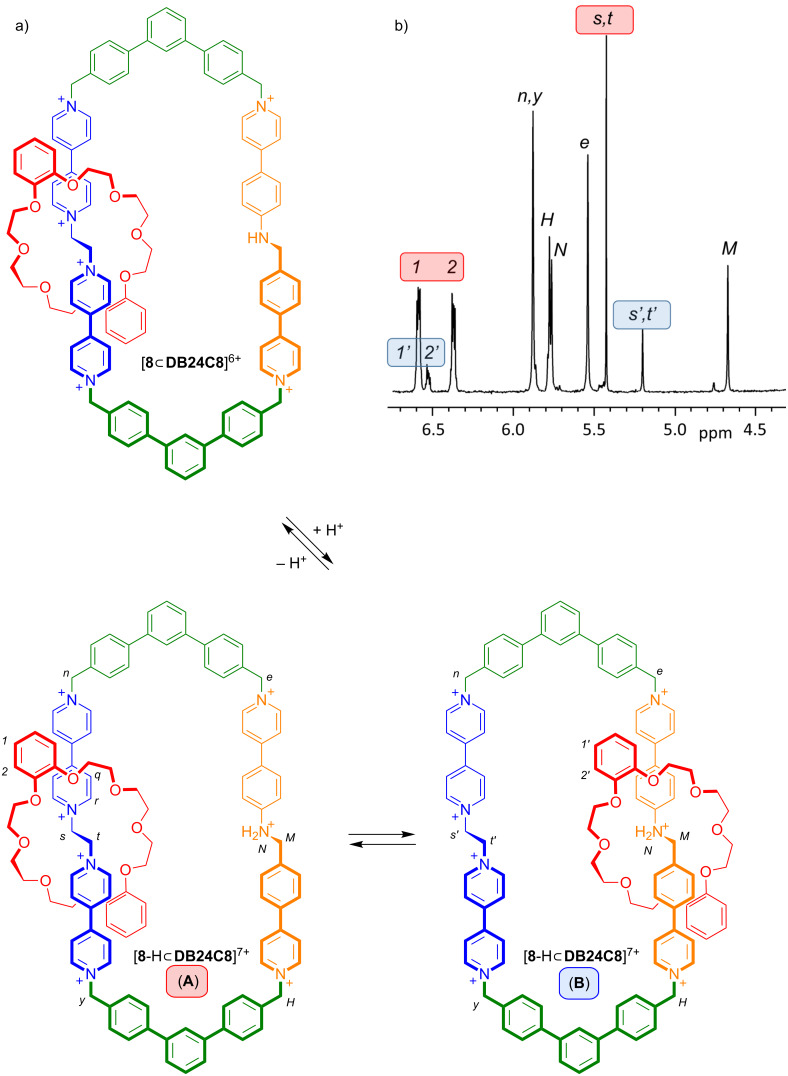
a) The [2]catenane [**8**
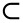
**DB24C8**]^6+^ can be protonated to yield [**8**-H
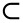
**DB24C8**]^7+^ in two different co-conformations **A** and **B**. b) The partial ^1^H NMR spectrum (500 MHz, 298 K, CD_3_CN) of [**8**-H
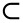
**DB24C8**]^7+^ shows key resonances for both co-conformations **A** (red) and **B** (blue). See Scheme 1 for labelling; atoms of co-conformer **B** are labelled with a prime, e.g., *s*’ versus *s*.

Figure 4 shows a partial ^1^H NMR spectrum of protonated [**8**-H
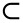
**DB24C8**]^7+^ in CD_3_CN at 298 K. The smaller **DB24C8** ring can now reside at either of the bis(pyridinium)ethane or benzylanilinium sites and these two possible co-conformations are designated **A** and **B** in Figure 4a. The ethylene protons at the core of the bis(pyridinium)ethane motif, labelled *s* and *t* in **A** and *s’* and *t’* in **B** are clearly distinguishable and show that there is a 4:1 ratio of **A**:**B** indicating that the smaller **DB24C8** ring prefers to occupy the bis(pyridinium)ethane site over the benzylanilinium site and that shuttling between the two sites is slow on the NMR timescale under these experimental conditions. Addition of base (NEt_3_) returns the system to its original state and the process can be cycled by repeated addition of acid (CF_3_SO_3_H) and base without significant degradation of the compound as verified by ^1^H NMR spectroscopy.

Interestingly, these results are contrary to those observed for the [2]rotaxane molecular shuttles [**1F**
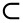
**DB24C8**]^2+^ and [**1A**
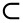
**DB24C8**]^2+^ shown in Figure 1. For that system, the benzylanilinium site was preferred 3:1 for [**1F**
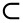
**DB24C8**]^2+^ and 9:1 for [**1A**
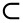
**DB24C8**]^2+^ in CD_3_CN and when CD_2_Cl_2_ was used the systems were completely bistable with **DB24C8** preferring to reside exclusively at the bis(pyridinium)ethane site when unprotonated and exclusively at the benzylanilinium site when protonated.

**Figure 5:**
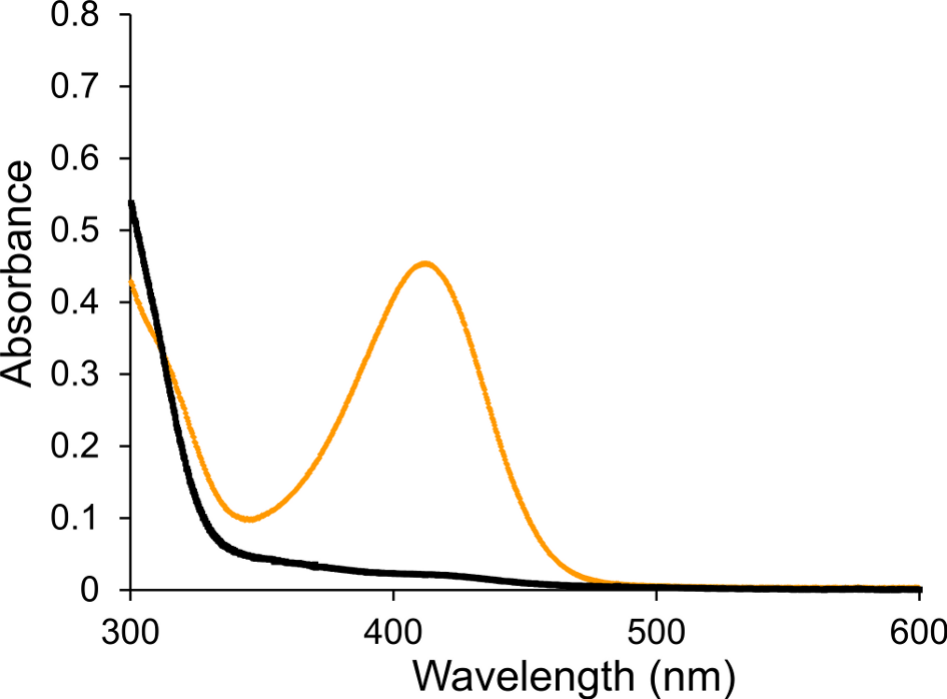
UV–visible spectra of [**8**
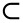
**DB24C8**]^6+^ (orange trace) and [**8**-H
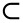
**DB24C8**]^7+^ (black trace) in CH_3_CN solution at 2.0 × 10^−5^ M and 298 K.

The UV–visible spectra of [**8**
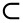
**DB24C8**]^6+^ and [**8**-H
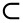
**DB24C8**]^7+^ are shown in Figure 5 for 2.0 × 10^−5^ M solutions in CH_3_CN. The molar absorptivity (ε) of [**8**
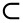
**DB24C8**]^6+^ was calculated to be 22,680 L mol^−1^ cm^−1^ with λ_max_ at 412 nm. The large absorption is due to an intramolecular charge transfer (ICT) band arising from charge transfer between the aniline nitrogen and pyridinium group of the benzylanilinium recognition site. However, this ICT band (412 nm) is eliminated by protonating the aniline nitrogen to form [**8**-H
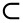
**DB24C8**]^7+^. Therefore, when the [2]catenane absorbs strongly showing a deep yellow/orange solution this indicates that the crown resides solely on the bis(pyridinium)ethane site for [**8**
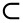
**DB24C8**]^6+^ but, when the [2]catenane does not absorb in the UV–visible region yielding a colorless solution this means the crown ether must be shuttling (i.e., circumrotating) back and forth between the two co-conformations, **A** and **B**, of [**8**-H
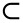
**DB24C8**]^7+^.

## Conclusion

A two-station circumrotational [2]catenane has been synthesized and its operation described. The system consists of a large macrocycle containing two different recognition sites, one bis(pyridinium)ethane and one benzylanilinium with a single smaller **DB24C8** ring that can shuttle between the two recognition sites depending on the protonation state of the larger macrocycle. When the aniline group is neutral, the **DB24C8** ring resides only at the bis(pyridinium)ethane site. However, addition of acid activates the benzylanilinium site allowing the ring to shuttle between the two, now competing, recognition sites. It was found that **DB24C8** prefers the bis(pyridinium)ethane site over the protonated benzylanilinium site in a ratio of 4:1. This is quite different from similar [2]rotaxane molecular shuttles (Figure 1) where, once protonated, the benzylanilinium site was preferred (CD_3_CN) and in some cases exclusively (CD_2_Cl_2_) generating a true ON/OFF bistable switch; unfortunately, the [2]catenane switch is insoluble in CD_2_Cl_2_ when protonated so a comparison could not be undertaken in this solvent. This difference in site populations between [2]rotaxane and [2]catenane is due to the presence of electron-withdrawing CF_3_ groups on the [2]rotaxane which make the benzylanilinium site more favorable in this case. Since it is fairly straightforward to change the nature of the stoppering groups of a [2]rotaxane dumbbell while the cyclic nature of the large ring makes it difficult to derivatize, [2]rotaxanes are deemed easier to fine-tune from a structural perspective than [2]catenanes. Although we were able to create an optically sensitive [2]catenane molecular shuttle with the bis(pyridinium)ethane and benzylanilinium recognition motifs, we could not achieve the true ON/OFF, bistable molecular switching previously observed for analogous [2]rotaxanes.

## Experimental

### General comments

4-Bromobenzyl bromide, 4-bromoaniline, 4-pyridylboronic acid, 1,3-dichlorobenzene, *p-*tolylmagnesium bromide, *n*-butyllithium and *N*-bromosuccinimide were purchased from Aldrich and used as received. Benzoyl peroxide was purchased from Acros and used as received. Compounds **3** [18], [**5**][OTf]_2_ [19–20] and **6** [21] were prepared using literature methods. Solvents were dried using an Innovative Technologies solvent purification system. Thin-layer chromatography (TLC) was performed using Teledyne Silica gel 60 F254 plates and viewed under UV light. Column chromatography was performed using Silicycle ultra-pure silica gel (230–400 mesh). The solvents were dried and distilled prior to use. NMR spectra were recorded on a Bruker Avance III console equipped with an 11.7 T magnet (e.g., 500 MHz for ^1^H). Samples were locked to the deuterated solvent and all chemical shifts reported in ppm referenced to tetramethylsilane. Mass spectra were recorded on a Waters Xevo G2-XS instrument. Solutions with concentrations of 0.001 molar were prepared in methanol and injected for analysis at a rate of 5 µL/min using a syringe pump.

#### Synthesis of **4**

DMF (250 mL) and H_2_O (100 mL) were added to a round bottom Schlenk flask (500 mL) and degassed with N_2_ for 2 h. To this solvent mixture, **3** (1.11 g, 0.00325 mol), 4-pyridylboronic acid (1.00 g, 0.00814 mol) and Na_2_CO_3_ (2.07 g, 0.195 mol) were added and the solution degassed for an additional 1 h. Catalyst [Pd(PPh_3_)_4_] (0.188 g, 16.3 mmol) was added and the solution degassed for an additional 30 min. The reaction was then refluxed for 5 days and the progress of the reaction monitored using ^1^H NMR spectroscopy. After the 5 days, the reaction was cooled to room temperature and the solvents removed by evaporation. The residue was dissolved in CH_2_Cl_2_ (100 mL) and washed with H_2_O (3 × 50 mL). The CH_2_Cl_2_ layer was dried over anhydrous MgSO_4_, filtered and concentrated. Compound **4** precipitated as a pale yellow powder which was collected by vacuum filtration. The filtrate was then evaporated under vacuum and the residue subjected to column chromatography (SiO_2_, 1% MeOH/CHCl_3_, *R*_f_ = 0.13) to yield further product. The batches of product (from precipitate and filtrate) were combined and recrystallized from acetone. Yield, 0.800 g, 73%; mp 186–188 °C; ^1^H NMR (500 MHz, CD_3_CN, 298 K) δ 8.60 (d, ^3^*J* = 6.1 Hz, 2H), 8.48 (d, ^3^*J* = 6.1 Hz, 2H), 7.71 (d, ^3^*J* = 8.2 Hz, 2H), 7.59 (d, ^3^*J* = 6.2 Hz, 2H), 7.54 (d, ^3^*J* = 8.7 Hz, 2H), 7.51 (d, ^3^*J* = 8.2 Hz, 2H), 7.49 (d, ^3^*J* = 6.2 Hz, 2H), 6.73 (d, ^3^*J* = 8.7 Hz, 2H), 5.41 (br t, 1H), 4.46 (d, ^3^*J* = 6.2 Hz, 2H); ^13^C NMR (125 MHz, CD_3_CN, 298 K) δ 151.1, 150.3, 149.8, 147.6, 146.8, 140.5, 140.2, 129.9, 128.4, 127.9, 127.6, 120.8, 120.4, 113.1, 47.6; HRMS (ESI) *m*/*z*: [M + H]^+^ calcd for [C_23_H_20_N_3_]^+^, 338.1657; found, 338.1650.

#### Synthesis of [**7**][OTf]_4_

[**5**][OTf]_2_ (0.400 g, 0.626 mmol) and **6** (2.61 g, 6.26 mmol) were dissolved in CH_3_CN (75 mL) and stirred at room temperature for 7 days. The resulting precipitate was filtered, collected and stirred in CH_2_Cl_2_ for 20 min and filtered to remove excess **6**. The precipitate was then anion exchanged to the triflate salt in a two-layer CH_3_NO_2_/NaOTf(aq) solution. The layers were separated and the CH_3_NO_2_ layer washed with H_2_O (3 × 5 mL) and then dried over anhydrous MgSO_4_. The CH_3_NO_2_ was removed by rotary evaporation and [**7**][OTf]_4_ isolated as a pale yellow solid. Yield 0.700 g, 69%; mp >180 °C (dec.); ^1^H NMR (500 MHz, CD_3_CN, 298 K) δ 9.05 (d, ^3^*J* = 6.9 Hz, 4H), 9.04 (d, ^3^*J* = 6.9 Hz, 4H), 8.50 (d, ^3^*J* = 6.1 Hz, 4H), 8.47 (d, ^3^*J* = 6.1 Hz, 4H), 7.91 (s, 2H), 7.86 (d, ^3^*J* = 8.2 Hz, 4H), 7.71 (d, ^3^*J* = 8.2 Hz, 4H), 7.68 (d, ^3^*J* = 7.9 Hz, 2H), 7.67 (d, ^3^*J* = 8.2 Hz, 4H), 7.62 (d, ^3^*J* = 8.0 Hz, 4H), 7.57 (t, ^3^*J* = 7.9, ^3^*J* = 8.1 Hz, 2H), 7.54 (d, ^3^*J* = 8.2 Hz, 4H), 5.89 (s, 4H), 5.30 (br s, 4H), 4.66 (s, 4H); HRMS (ESI) *m/z*: [M − OTf]^+^ calcd, 1457.1114; found, 1457.1144.

#### Synthesis of [**8**
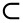
**DB24C8**][OTf]_6_

[**7**][OTf]_4_ (0.155 g, 0.0963 mmol) and **DB24C8** (0.432 g, 0.963 mmol) were dissolved in a two phase CH_3_NO_2_/H_2_O mixture and stirred at room temperature for 30 min to allow [2]pseudorotaxane formation. Compound **4** (0.0330 g, 0.0963 mmol) was then added along with NaOTf (0.0330 g, 0.193 mmol) and the reaction stirred at room temperature for 21 days. The water layer was separated and the CH_3_NO_2_ evaporated. The resulting residue was washed with CH_2_Cl_2_ (3 × 10 mL) to remove excess **DB24C8** and subjected to column chromatography on silica gel (5:3:2 mixture of CH_3_OH/NH_4_Cl (2 M)/CH_3_NO_2_). Fractions containing the product (*R*_f_ = 0.66) were combined and the solvents evaporated. The residue was dissolved in a two layer CH_3_NO_2_/NaOTf(aq) solution to anion exchange to the triflate salt. The H_2_O layer was removed and the CH_3_NO_2_ layer washed with H_2_O (3 × 5 mL) to extract any remaining salts. The CH_3_NO_2_ layer was dried with anhydrous MgSO_4_ and then evaporated to yield [**8**
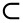
**DB24C8**][OTf]_6_ as a yellow-orange solid. Yield 0.030 g, 12%; mp >210 °C (dec.); HRMS (ESI) *m*/*z*: [M − 2OTf]^2+^ calcd for [C_113_H_103_F_12_N_7_O_20_S_4_]^2+^, 1116.7969, found, 1116.7972; [M − 3OTf]^3+^ calcd for [C_112_H_103_F_9_N_7_O_17_S_3_]^3+^, 694.8804, found, 694.8835; [M − 4OTf]^4+^ calcd for [C_111_H_103_F_6_N_7_O_14_S_2_]^4+^, 483.9222, found, 483.9246; [M − 5OTf]^5+^ calcd for [C_110_H_103_F_3_N_7_O_11_S]^5+^, 357.3472, found, 357.3465; ^1^H NMR (500 MHz, CD_2_Cl_2_, 298 K) δ 9.31 (d, ^3^*J_rq_* = 6.7 Hz, 2H, *r*), 9.31 (d, ^3^*J_uv_* = 6.7 Hz, 2H, *u*), 9.03 (d, ^3^*J_op_* = 6.8 Hz, 2H, *o*), 9.03 (d, ^3^*J_xw_* = 6.8 Hz, 2H, *x*), 8.76 (d, ^3^*J_IJ_* = 6.8 Hz, 2H, *I*), 8.49 (d, ^3^*J_dc_* = 6.9 Hz, 2H, *d*), 8.22 (d, ^3^*J_qr_* = 6.7 Hz, 2H, *q*), 8.22 (d, ^3^*J_vu_* = 6.7 Hz, 2H, *v*), 8.22 (d, ^3^*J_JI_* = 6.8 Hz, 2H, *J*), 8.19 (d, ^3^*J_po_* = 6.8 Hz, 2H, *p*), 8.19 (d, ^3^*J_wx_* = 6.8 Hz, 2H, *w*), 8.04 (d, ^3^*J_cd_* = 6.9 Hz, 2H, *c*), 7.87 (d, ^3^*J_lm_* = 8.2 Hz, 2H, *l*), 7.87 (d, ^3^*J_Az_* = 8.2 Hz, 2H, *A*), 7.87 (d, ^3^*J_KL_* = 8.2 Hz, 2H, *K*), 7.83 (s, 1H, *k*), 7.83 (s, 1H, *E*), 7.80 (d, ^3^*J_gf_* = 8.4 Hz, 2H, *g*), 7.78 (d, ^3^*J_ba_* = 8.7 Hz, 2H, *b*), 7.77 (d, ^3^*J_FG_* = 8.6 Hz, 2H, *F*), 7.74–7.70 (d, 1H, *h*), 7.74–7.70 (d, 1H, *j*), 7.74–7.70 (d, 1H, *B*), 7.74–7.70 (d, 1H, *D*), 7.64 (d, ^3^*J_ml_* = 8.2 Hz, 2H, *m*), 7.64 (d, ^3^*J_zA_* = 8.2 Hz, 2H, *z*), 7.62 (dd, 1H, *i*), 97.62 (dd, 1H, *C*), 7.58 (d, ^3^*J_LK_* = 8.2 Hz, 2H, *L*), 7.55 (d, ^3^*J_GF_* = 8.6 Hz, 2H, *G*), 7.51 (d, ^3^*J_fg_* = 8.4 Hz, 2H, *f*), 6.79 (d, ^3^*J_ab_* = 8.7 Hz, 2H, *a*), 6.62 (m, ^3^*J_ortho_* = 5.8, ^3^*J_meta_* = 3.6 Hz, 4H, *1*), 6.33 (m, ^3^*J_ortho_* = 5.8, ^3^*J_meta_* = 3.6 Hz, 4H, *2*), 5.90 (s, 2H, *n*), 5.90 (s, 2H, *y*), 5.87 (t, ^3^*J_NM_* = 5.6 Hz, 1H, *N*), 5.74 (d, 2H, *H*), 5.59 (s, 2H, *e*), 5.56 (s, 2H, *s*), 5.56 (s, 2H, *t*), 4.48 (d, ^3^*J_MN_* = 5.6 Hz, 2H, *M*), 4.04–3.99 (m, 24H, *3–5*).
